# Identifying Risk Zones for Neurovascular Injury in Pediatric All-Inside Arthroscopic Lateral Meniscal Repair

**DOI:** 10.1177/23259671241304817

**Published:** 2025-03-03

**Authors:** Annat Houston, Casey McDonald, Andrew Eck, Travis Kotzur, David Momtaz, David Heath, Grant D. Hogue, Thomas DeBerardino

**Affiliations:** † Department of Orthopedic Surgery, University of Texas Health Science Center at San Antonio, San Antonio, Texas, USA; ‡ Long School of Medicine, University of Texas Health Science Center at San Antonio, San Antonio, Texas, USA; § Department of Orthopedic Surgery, Boston Children’s Hospital, Boston, Massachusetts, USA; Investigation performed at the University of Texas Health Science Center at San Antonio, San Antonio, Texas, USA

**Keywords:** all-inside, meniscal repair, arthroscopy

## Abstract

**Background:**

All-inside techniques for meniscal repairs offer comparable outcomes and healing rates with reduced operative time and fewer incisions; however, iatrogenic neurovascular injuries during arthroscopic meniscal repairs are a significant concern.

**Purpose:**

To identify the zones of risk and incidence of injury concerning the common peroneal nerve (CPN) and popliteal artery in relation to the popliteal tendon (PT) from the anterolateral (AL) and anteromedial (AM) portals during a simulated all-inside technique in the pediatric population.

**Study Design:**

Descriptive laboratory study.

**Methods:**

Using axial knee magnetic resonance imaging scans of 124 patients, the all-inside technique was simulated by drawing direct lines from the AM and AL portals to the medial and lateral borders of the PT. If the line came into contact with the CPN, a risk of projected iatrogenic CPN injury was found. Measurements were then recorded to assess and define “risk zones.” A similar simulation was performed in relation to the popliteal artery to assess distance to projected iatrogenic injury.

**Results:**

The risk of CPN injury was significantly higher when using the AL portal (45%) compared with the AM portal (19%) when simulating repair at the lateral edge of the PT (*P* < .001). Similarly, there was a significantly higher risk of peroneal nerve injury when using the AM portal (29%) compared with the AL portal (8.9%) when simulating repair from the medial edge of the PT (*P* < .001). The risk of injury when repairing the body of the lateral meniscus through the AM portal extended 2.20 ± 0.98 mm laterally from the lateral edge of the PT and 3.14 ± 1.92 mm medially from the medial edge of the PT. The risk of injury when repairing the body of the lateral meniscus through the AL portal extended 2.58 ± 1.31 mm lateral to the lateral edge of the PT and 2.02 ± 1.61 mm medial to the medial edge of the PT.

**Conclusion:**

The authors found that the AM portal was safer for repairing the body of the lateral meniscus while simulating repair at the lateral edge of the PT, while the AL portal was safer for repairing the lateral meniscus while simulating repair from the medial edge of the PT.

**Clinical Relevance:**

By understanding these risk profiles, surgeons can adopt safer approaches for meniscal repairs in pediatric patients, thereby minimizing the likelihood of injuring sensitive neurovascular structures.

Over the past several decades, there has been a significant increase in sports participation among the pediatric population. The increase in sports participation is thought to cause an increased rate in meniscal injury.^7,28^ As described in previous literature, pediatric meniscal injuries were thought to be rare and were specifically associated with embryological malformations such as discoid meniscus.^21^ However, with advanced imaging (eg, magnetic resonance imaging [MRI]) as well as improved technology with arthroscopic tools, more meniscal injuries are being found in the pediatric population. Notably, pediatric patients with a high body mass index (BMI), year-round sports activity with excessive pivoting mechanisms, or concomitant anterior cruciate ligament injuries have been associated with higher risk of meniscal tears in children.^5^ Peripheral tears have been shown to most likely heal, while vertical tears are most likely to propagate. The ability to heal the meniscal injuries matches the “red and white zones” of vascularity as described in adult patients.^2,18,22^

In order to maintain and preserve native knee anatomy and biomechanics, meniscal repair is crucial, as it helps to reduce excessive stress in the knee joint.^28^ Various techniques have been employed for meniscal repairs, including outside-in, inside-out, and all-inside suture techniques. While the inside-out technique has traditionally been considered the gold standard for meniscal repair, recent studies have demonstrated that all-inside techniques offer comparable functional outcomes and healing rates, with reduced operative time and fewer incisions.^14^ Specifically, the all-inside technique has become more popularly utilized for vertical and radial tears and posterior horn tears of both the medial and the lateral menisci.

When performing posterolateral meniscal repairs, capsular anchor points are ideally placed on either side of the popliteal tendon (PT) to avoid penetrating the tendon itself. Additionally, neurovascular structures lie on either side, including the common peroneal nerve (CPN) and the popliteal artery. Iatrogenic neurovascular injuries during arthroscopic meniscal repairs involving the CPN have been reported in several case studies.^3,19,20^ Some studies have reported complications around 1.7% to 1.8% and others from 1% to 8% depending on type of iatrogenic injury.^15,29^ The injuries often result from accidental trapping or tethering of the nerve during suture placement. Although rare, injuries to the popliteal artery have also been documented,^8^ with the incidence being <1%.^26^ Numerous studies, including cadaveric and diagnostic imaging investigations, have explored iatrogenic injuries to the peroneal nerve due to its close proximity to the PT at the level of the lateral joint line.^4,6,9,31^

In a recent MRI study, Chuaychoosakoon et al^11^ established “danger zones” and “safe zones” for iatrogenic neurovascular injuries in the adult population while simulating the all-inside technique. Their findings indicated that the anterolateral (AL) portal was more dangerous to use when repairing the lateral meniscus beyond the lateral edge of the PT, with a potential injury rate to the CPN being 14%. Additionally, when using the all-inside device at the medial border of the PT, there was a 20% potential injury rate to the CPN when instrumenting from the anteromedial (AM) portal.^11^

In the current study, we aimed to address the “zones of risk” in the pediatric population in relation to repairing the lateral meniscus, considering the developing and growing musculoskeletal structures in this patient population. Specifically, we wanted to evaluate the proximity of the peroneal nerve with incidence rates of potential CPN injury as well as proximity of the popliteal artery while simulating the all-inside technique from the AL and AM portals via MRI studies. Additionally, we assessed several variables, including BMI, age, and sex, to determine their effects on these zones of risk. We hypothesized that an increase in BMI would decrease the risk of injury to the CPN.

## Methods

### Study Protocol

The protocol for this study was determined to be exempt from needing institutional review board approval. A retrospective analysis was performed on patients aged 8 to 18 years who had undergone MRI of the knee at our institution between 2008 and 2020. Inclusion criteria were the availability of a completed, reviewable MRI and no prior history of knee surgery. Exclusion criteria were patients with evidence of anterior or posterior cruciate ligament injury to prevent any potential bias in anterior or posterior translation measurements, incomplete data available for collection, or poor-quality imaging.

Two senior-level orthopaedic residents (A.H., A.E.) evaluated the MRI scans and assessed the distances in relation to the AL and AM portals to the sites of interests discussed below. Measurements were then finalized based on consensus.

### Image Analysis

**Figure 1.**
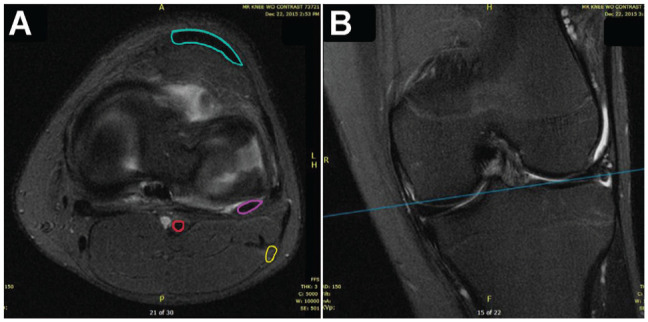
(A) Axial and (B) coronal side-by-side MRI sequences at the level of the lateral meniscus. Line in (B) is the correlated level of the axial image in A. Outlined in (A) are the patellar tendon (blue), popliteal tendon (purple), common peroneal nerve (yellow), and popliteal artery (red).

**Figure 2.**
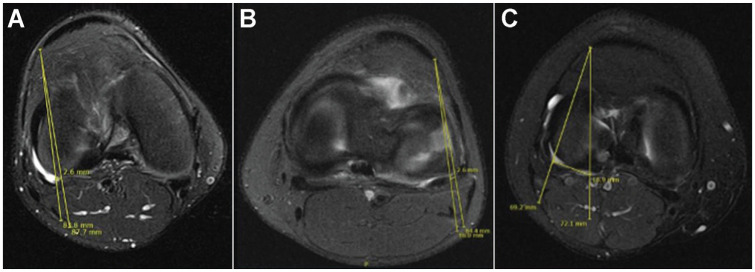
Simulating the anterolateral (AL) approach of the all-inside suture repair device on axial magnetic resonance imaging scans. The lines in (A) and (B) are drawn from the simulated all-inside device entering from the AL portal to the medial and lateral edges of the popliteal tendon (PT) and the calculated distance from the respective borders of the peroneal nerve. (C) demonstrates the simulated all-inside device entering from the AL portal to the medial edge of the PT and the calculated distance from the popliteal artery.

**Figure 3.**
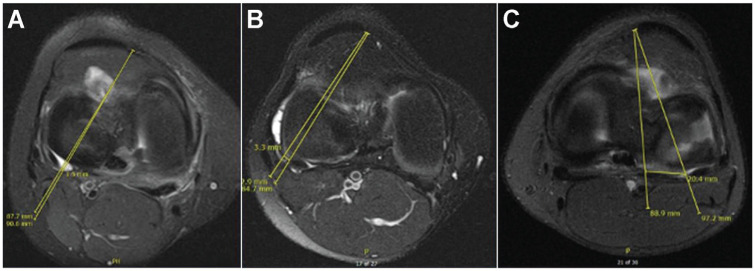
Simulating the anteromedial (AM) approach of the all-inside suture repair device on axial magnetic resonance imaging scans. (A) and (B) are lines drawn from the simulated all-inside device entering from the AM portal to the medial and lateral edges of the popliteal tendon (PT) and the calculated distance from the respective borders of the peroneal nerve. (C) demonstrates the simulated all-inside device entering from the AM portal to the medial edge of the PT and the calculated distance from the popliteal artery.

The protocol used in this pediatric study was first established by Chuaychoosakoon et al^11^ in their MRI study of adult knees. Axial and coronal MRI sequences were displayed side-by-side to confirm the level of the lateral meniscus, as shown in Figure 1. Notably, the MRI scans were obtained with the patient lying supine with the knee slightly flexed. The medial and lateral borders of the patellar tendon were designated as the AM and AL portals, respectively, with the height of each portal being at the level of the lateral meniscus seen on axial MRI. This was to create a standard point to reduce heterogeneity with the measurements. To simulate the all-inside suture repair device, a line was drawn from the AL portal to the lateral edge of the PT and was extrapolated beyond the capsule to determine if it violated the CPN territory. (A depth on the extrapolation was not used, as the all-inside device may be set to variable depths that can range from 8 to 18 mm.^23^) A risk of iatrogenic injury was determined to exist if this line intersected the CPN. If the CPN was contacted or intersected, another line was drawn from the AL portal site to the lateral border of the CPN. The distance along the meniscocapsular junction between these 2 lines was measured and calculated as the zone of risk (Figure 2). This process was repeated with the relationship between the AL portal and the medial border of the PT. The same lines and distances were then evaluated in relation to the AM portal (Figure 3). For extrapolated lines drawn from the portal to the edge of the PT (medial or lateral) that did not contact the peroneal nerve, these data points were excluded from the calculation but were noted as being in the no-risk zone.

To assess the risk of injury to the popliteal artery, lines were drawn from the AL portal to the medial border of the PT and from the AL portal to the lateral edge of the popliteal artery. The distance between these 2 lines was measured along the meniscocapsular junction. The same process was performed in relation to the AM portal (Figures 2C and 3C). All patients were included in the calculation of risk injury to the popliteal artery.

### Statistical Analysis

A prospective study performed by Calfee et al^10^ demonstrated that mean skeletal age of maturity for boys was 15.3 ± 2.1 and 15.0 ± 2.0 for girls.^17,25^ As a result, we divided the study participants into skeletally immature (<15 years) and skeletally mature (≥15 years) groups. Once the individual groups were established, a comparative analysis was carried out to evaluate the distance, odds of crossing critical structures, and means of various variables between the groups. The categorical results were expressed as counts with column percentages, and continuous data were reported as means with standard deviations. Prior to performing statistical tests, all data underwent preliminary analysis to confirm that the statistical approach chosen was suitable and that the variables met the required assumptions for each test. Independent-samples *t* tests were employed for comparing normally distributed data, whereas the Wilcoxon rank-sum test was used for nonnormally distributed data. Categorical variables were assessed with the Fisher exact test or Pearson chi-square test, as appropriate.

Mixed-effects linear and logistic regression models were employed to investigate the association between the side of approach and the likelihood of encountering a critical neurovascular structure, while incorporating patient-specific random effects to account for the fact that each patient’s MRI was used to model both approaches. This modeling framework allowed for the assessment of intrapatient variability and controls for confounding variables including age, sex, ethnicity, race, and BMI *z* score, thereby enhancing our understanding of the impact of surgical approach while acknowledging the repeated measures within patients.

The multiple linear and logistic regression models were scrutinized to ensure the fulfillment of all underlying assumptions. The normal distribution of residuals was assessed, and multicollinearity was ruled out where applicable. All variables included in the models were individually assessed to verify the absence of artifact *P* values and to ensure transparent reporting of effect sizes.

The statistical and power analyses were performed using the R Statistical Software, Version 4.3.1 (R Core Team). All results from regression analyses are reported as odds ratios (ORs) along with their 95% CIs. A *P* value of <.05 was considered statistically significant.

## Results

### Population Characteristics

A total of 124 patients were included in this study (42 patients aged <15 years and 82 patients aged ≥15 years), with a mean age of 15 ± 2 years. The demographic breakdown of the study participants can be seen in Table 1. There was a significant difference between the age groups in BMI: 24.92 ± 6.99 for patients aged <15 years compared with 27.94 ± 6.01 for those aged ≥15 years (*P* = .008). The overall *z* score for BMI after adjusting for sex and age was 1.34 ± 0.94 (1.24 ± 1.09 for patients <15 years and 1.40 ± 0.84 for those ≥15 years).

**Table 1:** Patient Characteristics and Common Peroneal Nerve Injury Incidence^*a*^

Variable	Overall	Age <15 y	Age ≥15 y	*P*
	(N = 124)	(n = 42)	(n = 82)	
Patient characteristics				
Age, y	14.96 ± 2.15	12.48 ± 1.50	16.23 ± 1.02	**.001**
Sex, female	42 (33.9)	20 (47.6)	22 (26.8)	.05
Race, Black	9 (7.3)	1 (2.3)	8 (9.8)	.15
Ethnicity, Hispanic	76 (61.3)	25 (59.5)	51 (62.2)	.20
BMI	26.81 ± 6.52	24.92 ± 6.99	27.94 ± 6.01	**.008**
BMI *z* score^*b*^	1.34 ± 0.94	1.24 ± 1.09	1.40 ± 0.84	.40
Peroneal nerve injury				
From lateral border of PT to edge of CPN				
From AL portal	56 (45.2)^*c*^	24 (57.1)	32 (39.0)	.06
From AM portal	24 (19.4)^*c*^	8 (19.0)	16 (20.0)	.90
From medial border of PT to edge of CPN				
From AL portal	11 (8.9)^*c*^	6 (14.3)	5 (6.1)	.20
From AM portal	36 (29.0)^*c*^	13 (31.0)	23 (28.0)	.70

a Data are presented as mean ± SD or n (%). Boldface *P* values indicate statistically significant difference between age groups (*P* < .05). AL, anterolateral; AM, anteromedial; BMI, body mass index; CPN, common peroneal nerve; PT, popliteal tendon.

b *z* score adjusted for sex and age.

c Significant difference between AL and AM portal (*P* < .001).

### Incidence of CPN Injury

**Figure 4.**
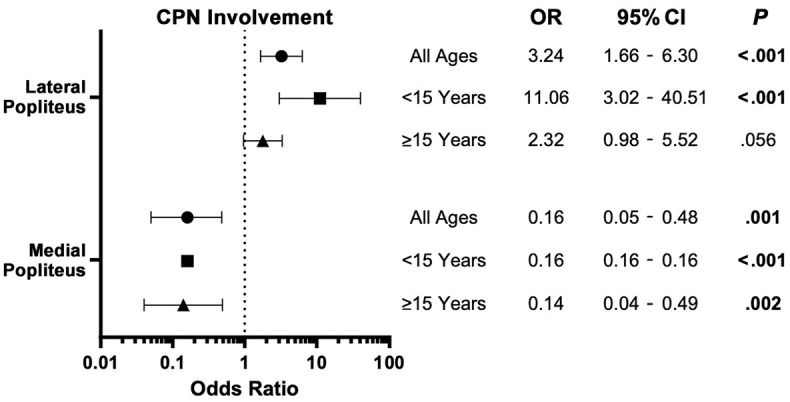
Forest plot depicting the odds ratio (OR) after multivariate regression of crossing the common peroneal nerve (CPN) from the anterolateral portal versus the anteromedial portal for both lateral and medial popliteal approach. Data points are stratified by age groups: all ages, <15 years, and ≥15 years, with error bars indicating 95% CIs. For both linear and log transformations, 95% CIs and *P* values are provided. Boldface *P* values indicate statistical significance (*P* < .05).

**Figure 5.**
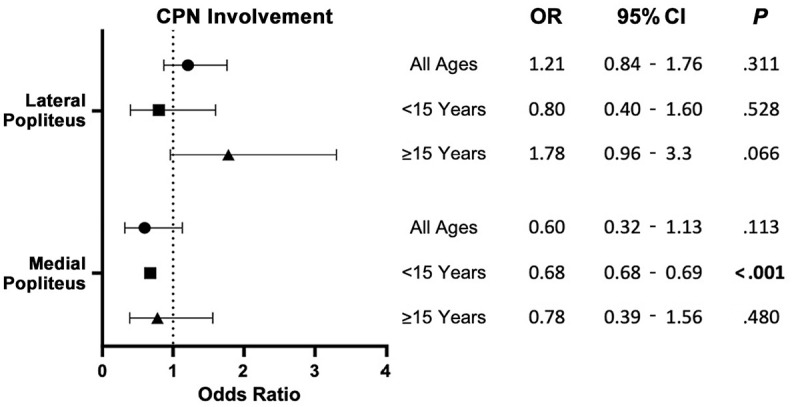
Forest plot depicting the odds ratio (OR) associated with each change in the *z* score for BMI on the likelihood of affecting the common peroneal nerve (CPN). Data are stratified by age group: all ages, <15 years, and ≥15 years. Each point on the graph represents the OR, with error bars indicating 95% CIs. Boldface *P* value indicates statistical significance (*P* < .05).

The risk of CPN injury was significantly higher (*P* < .001) at the lateral edge of the PT when instrumenting from the AL portal (45%) versus the AM portal (19%) (Table 1). The inverse was found to be true at the medial border of the PT, with a significantly higher risk (*P* < .001) of CPN injury when instrumenting from the AM portal (29%) compared with from the AL portal (8.9%). The same trend was found in both the patients aged <15 years and those aged ≥15 years. When approaching the lateral edge of the popliteus from the AL border, there was a 3.24 times increased odds of crossing the CPN compared with using the AM portal. In patients <15 years old, the OR increased to 11.06 in this scenario (Figure 4). In the multivariate analysis, BMI was not found to have a significant effect on the probability of crossing the CPN for most age and approach categories. However, a notable exception was observed in the patients aged <15 years using the medial approach to the PT (Figure 5). For this demographic, each unit increase in BMI *z* score above the median was associated with a 32% reduction in the odds of CPN involvement (OR, 0.68; 95% CI, 0.68-0.69; *P* < .001).

### Nerve Zones of Risk

In our study, we observed the danger zone for each portal in relation to the PT borders. Approaching from the AL portal, the risk of injury to the CPN extended 2.58 ± 1.31 mm laterally from the lateral edge of the PT and 2.02 ± 1.61 mm medially from the medial edge of the PT. Approaching from the AM portal, the risk of injury to the CPN extended 2.20 ± 0.98 mm laterally from the lateral edge of the PT and 3.14 ± 1.92 mm medially from the medial edge of the PT. All of these findings indicated significant differences between the AL and AM portal approaches with respect to the edges of the PT (P < 0.001 for all) (Table 2). These findings highlight the varying risk profiles associated with different portal approaches and their relation to the PT borders in pediatric patients.

**Table 2:** Distances to Nerve Injury in Relation to Popliteal Edge and Portal Site^*a*^

Distance, mm	All Patients		Age <15 y		Age ≥15 y	
	Mean ± SD	*P*	Mean ± SD	*P*	Mean ± SD	*P*
From lateral edge of PT to edge of CPN		**<.001**		**<.001**		.07
From AL portal	2.58 ± 1.31		2.50 ± 1.47		2.64 ± 1.19	
From AM portal	2.20 ± 0.98		1.88 ± 0.64		2.37 ± 1.09	
From medial edge of PT to edge of CPN		**<.001**		**<.001**		**.04**
From AL portal	2.02 ± 1.61		1.67 ± 1.21		2.38 ± 1.99	
From AM portal	3.14 ± 1.92		3.29 ± 2.16		3.05 ± 1.80	
From medial edge of PT to popliteal artery		**<.001**		**<.001**		**<.001**
From AL portal	15.55 ± 4.39		15.65 ± 4.62		15.51 ± 4.29	
From AM portal	18.78 ± 4.32		18.75 ± 4.33		18.80 ± 4.34	

a Boldface *P* values indicate statistically significant difference between AL and AM approaches for that measurement (*P* < .05; paired *t* test). AL, anterolateral; AM, anteromedial; CPN, common peroneal nerve; PT, popliteal tendon.

When we divided our population into age groups of <15 years (skeletally immature) and ≥15 years (skeletally mature), we found that for the skeletally immature patients, when approaching from the AL portal the danger zone extended 2.50 ± 1.47 mm laterally from the lateral edge of the PT and 1.67 ± 1.21 mm medially from the medial edge of the PT. When approaching from the AM portal, the danger zone extended 1.88 ± 0.64 mm laterally from the lateral edge of the PT and 3.29 ± 2.16 mm medially from the medial edge of the PT. These findings demonstrated significant differences between approaches (AL vs AM portal) with respect to the particular edge of the PT (*P* < .001 for all) (Table 2). For the skeletally mature patients, when approaching from the AL portal, the danger zone extended 2.64 ± 1.19 mm laterally from the lateral edge of the PT and 2.38 ± 1.99 mm medially from the medial edge of the PT. Approaching from the AM portal, the danger zone extended 2.37 ± 1.09 mm laterally from the lateral edge of the PT and 3.05 ± 1.80 mm medially from the medial edge of the PT. Significant differences between the portal approaches were seen in the PT borders from the medial edge of the PT both to the edge of the CPN (*P* = .04) and to the popliteal artery (*P* < .001).

### Artery Zones of Risk

The risk of injury to the popliteal artery began 18.78 ± 4.32 mm from the medial edge of the PT when approaching from the AM portal. When approaching from the AL portal, the risk of injury to the popliteal artery began 15.55 ± 4.39 mm from the medial edge of the PT (Table 2), which demonstrated statistical significance. We also found a significant difference with similar distances to the popliteal artery within the skeletally mature and immature subgroups.

## Discussion

In this study, we evaluated knee MRIs in pediatric patients to characterize and establish “danger zones” in relation to the AM and AL portals used during all-inside lateral meniscal repair. By utilizing MRI imaging on pediatric patients, we were able to define these danger zones similar to the study by Chuaychoosakoon et al^11^ in the adult population. In analyzing pediatric patients’ MRIs, we found that the AM portal in comparison with the AL portal is safer for repairing the body of the lateral meniscus near the lateral edge of the PT with a lower risk of injury to the CPN (19%), while the AL portal in comparison with the AM portal is safer for repairing the body of the lateral meniscus near the medial edge of the PT with a lower risk of injury to the CPN (8.9%). However, when comparing both approaches and both borders of the PT, the AL portal posed the highest risk at injuring the CPN near the lateral edge of the PT with a potential injury rate of 45%. These results align with the study by Chuaychoosakoon et al and another MRI study in adults^30^ that have reported similar findings. Subsequent research by Boonsri et al^9^ further supported this observation in adults by examining knee positions that more closely resembled actual surgical scenarios. Additionally, we found that these results and conclusions stayed consistent when our pediatric population was subdivided into mature and skeletally immature.

In the current study, BMI was not found to have a significant effect on the probability of contacting the CPN for most age and approach categories. However, an exception was found in the skeletally immature patients in which an increase in the *z* score for BMI was associated with a reduction in CPN injury. We also concluded that the skeletally mature subgroup with a higher mean BMI of 27.94 and BMI *z* score of 1.40 had lower rates of CPN injury in comparison with the skeletally immature group with a mean BMI of 24.92 and BMI *z* score of 1.24; however, the difference in BMI *z* score was not statistically significant. This could be attributable to smaller knee circumference and less adipose with tighter structures, allowing for higher risk of penetration.

Our study offers a unique contribution to existing literature, as it is the first pediatric MRI investigation that examines the proximity of the peroneal nerve and popliteal artery relative to the AL and AM portal sites while simulating the all-inside meniscal repair technique and device. Notably, in our study in pediatric patients, we did find higher theoretical rates of peroneal nerve injury compared with Chuaychoosakoon et al’s^11^ study in adults. This difference may be due to the smaller dimensions of the pediatric knee, which increase the proximity of neurovascular structures and, consequently, the risk of injury during meniscal repair.^13,24,31^ However, the figure-of-4 and saline-dilated position analyzed by Boonsri et al^9^ in their MRI study demonstrated an incidence of 69% with repair of the lateral meniscus through the AL portal in relation to the lateral border of the PT. Our results mirror this and demonstrated that all pediatric patients had an incidence of 45%. This increased to 57% for patients who were aged <15 years.

When comparing our findings with the studies on adults,^9,11^ we observed consistent risk profiles across different portal approaches and their relation to the PT border in our pediatric population. Instrumenting from the AL portal posed less of a risk of iatrogenic injury to the CPN when repairing the posterolateral meniscus near the medial border of the PT, and instrumenting from the AM portal posed less of a risk of iatrogenic injury to the CPN when repairing the posterolateral meniscus near the lateral border of the PT. This knowledge serves as a valuable guide for surgeons performing surgical procedures on pediatric patients who have similar adult concepts in mind. Additionally, our pediatric-specific zones of risk share some similarities with adult data but also exhibit differences. Pediatric patients that are ≥15 or skeletally mature have a lower risk in comparison with those <15 years, so extra care should be undertaken, as the risk of injury is increased. By understanding these risk profiles, surgeons can adopt safer approaches for meniscal repairs in pediatric patients, thereby minimizing the likelihood of injuring sensitive neurovascular structures.

The distance to the popliteal artery is a critical consideration during meniscal repair in the pediatric population. Our findings emphasize the importance of being aware of the spatial relationships between the popliteal artery and surrounding structures to minimize the risk of injury during surgery. By maintaining an understanding of the popliteal artery’s location in relation to the portals and PT borders, surgeons can make safer surgical decisions and improve patient outcomes.

### Limitations

This study has several limitations. Our MRI measurements were performed with the knee in slight flexion as patients were lying down. A cadaveric study by Cuellar et al^12^ demonstrated that neurovascular risk is significantly lower when repairing the knee in flexion due to increased distances between the meniscocapsular junction and neurovascular structures. However, Boonsri et al^9^ conducted a study using a similar simulation technique on knee MRIs performed in the figure-of-4 position with fluid dilated knees, which more accurately reflects the knee’s position during arthroscopic repair of the lateral meniscus. Their research demonstrated high incidence rates (69%) of CPN injury along the lateral border of the PT while instrumenting from the AL approach. Although our study evaluated MRIs with the knee in minimal flexion, their research indicated that the position of the knee did not substantially affect the incidence rates of CPN injury while instrumenting from the AL portal at the lateral border of the PT.

Previous research has recommended penetration depths ≤18 mm to prevent popliteal neurovascular bundle injury, and not <8 mm to ensure repair integrity and a ratio of <0.05 (penetration depth: measured circumference of the knee) to avoid injury to the popliteal neurovascular bundle or the CPN.^1,16^ We did not specifically evaluate penetration depth in the current study, which is a notable limitation. However, as the penetration depth continues to change with varying instrumentation and techniques, we chose to focus on identifying whether these neurovascular structures were in the trajectory of a potential repair. This also allows for extrapolation of our data to other repair techniques.

## Conclusion

Future research should include patients with the CPN immediately posterior to the PT, rather than excluding them, as this may provide additional insights into the risk factors associated with this anatomic variant.^27^ Further studies should also consider evaluating knee positions that more closely resemble actual surgical scenarios, such as the figure-of-4 position, to enhance the clinical relevance of the findings. Additionally, repeating the study in anterior or posterior cruciate deficient knees would be of benefit, as many surgeons may prefer to perform meniscal repair before cruciate ligament reconstruction. By expanding our understanding of the pediatric-specific zones of risk and their implications on surgical outcomes, we can continue to refine and improve surgical techniques, ultimately leading to better patient outcomes and reduced rates of iatrogenic injury.
